# Planes in Aesthetic Breast Surgery: Is Subfascial a Misnomer?

**DOI:** 10.1093/asjof/ojae107

**Published:** 2024-11-10

**Authors:** Allen Gabriel, Erin N Abbott, Patrick Maxwell, Steven Sigalove, Galen Perdikis

Breast augmentation is the most popular breast surgery, with The Aesthetic Society reporting ∼250,000 augmentation procedures in 2023, an 11% increase since 2019.^1^ With the advances in implant technology, soft tissue support, and surgical technique, we have seen a resurgence of “subfascial” augmentation. A systematic review in 2024 compared outcomes and complications of subfascial and subglandular pockets. They found subfascial pocket had fewer complications, albeit with relatively short follow-up time, and importantly reported a high risk of bias due to poor randomization in the included studies.^2^

Various dissection planes for implant placement have been described throughout the evolution of breast augmentation, allowing for colorful commentary from leaders in the field. The main planes outlined in the literature include subglandular, subfascial, and subpectoral, all named for the location relative to the pectoralis major, as well as dual plane, which is partially subpectoral. The pectoralis major originates on the medial half of clavicle, sternum, fifth to seventh ribs, and external oblique muscles, then fanning laterally over the anterior chest to insert on the proximal humerus. The thickness of the pectoral fascia has been reported to range from 0.1 to 1.1 mm,^3,4^ providing a basis for debate on the use of the subfascial pocket. The subpectoral plane is posterior to the pectoralis major, while the subglandular plane is anterior to the muscle and its fascia, posterior to the breast tissue anchored to the fascia with Cooper's ligament. The subfascial approach aims to dissect between the pectoralis major muscle and its fascia, leaving only a thin, collagenous layer to support the implant long term.

Given our collective experience in aesthetic breast surgery, we argue against the existence of a true subfascial surgical plane. Ultimately, we will recommend the term “prepectoral” in its place to encompass both “subfascial” and subglandular planes, reflecting a more accurate anatomical term for this pocket. This will allow for future data collection to be more streamlined and accurate.

Historically, subglandular placement was the predominant approach for breast augmentation, where implants were placed in a prepectoral plane. Dissection in the subglandular plane was blunt and quite inaccurate, inevitably leaving some tissue on the pectoralis fascia. Due to the quality of both the implants and surgical techniques at the time, capsular contracture rates were high, reported to be between 15% and 45%.^5^ As the silicone moratorium transpired in the early 1990s, plastic surgeons were concurrently rethinking implant placement in the prepectoral plane. As saline implants became more widely adopted, common practice shifted from prepectoral to subpectoral to camouflage their over-filled roundness. However, this trend of total muscle coverage subsequently led to high riding implants and waterfall deformities, or “snoopy breast.”

To address these deformities, the dual plane pocket was later introduced to enhance the upper pole with soft tissue from the pectoralis major; this remains a mainstay pocket option today with ∼80% of surgeons using it. Tebbetts popularized and codified the dual plane and TEPID system, emphasizing the importance of soft tissue coverage for maintaining long-term augmentation aesthetics, not just reducing long-term capsular contracture.^4^

Trans-axillary dissection and implant placement were first popularized by Hoehler in 1973. Through a series of 228 patient cases, he anecdotally reported similar cosmetic outcomes to an inframammary fold (IMF) approach.^3^ Graf, another proponent of the trans-axillary approach, noted thicker prepectoral fascia through her experience with the Brazilian patient population.^3^ Subsequently, she introduced the concept of “subfascial” implant placement, or a “subfascial” pocket. Tebbetts ardently opposed this idea at the time, and, in future commentary, argued for the selection of a pocket with maximal tissue thickness and size for long-term device coverage, criteria not necessarily guaranteed with Graf's subfascial pocket.^4^

Today, with advanced surgical techniques and higher precision, the subglandular plane is approached quite differently across the globe. In Europe and South America, there is a higher usage of the subfascial plane, likely correlating with their higher rates of a trans-axillary approach. In contrast, using the IMF approach, in our experience, there is significant difficulty in visualizing and dissecting the thin, inferior fascial tissue to create a subfascial plane with an IMF incision. Nevertheless, the pectoral fascia can inadvertently be included. On the contrary, a subfascial dissection may end up with a portion, or even majority, of the pocket unintentionally in the subglandular plane.

Recently, we have seen increased discussion on subfascial implant placement, but it is crucial to deliberate: *is there a true subfascial plane?* Of course, anatomically, the plane exists. We can see the pectoral fascia and the dissection plane well with a sharp, clean cadaveric dissection. However, in the operating room, this scenario often unfolds quite differently.

The pectoral fascia is thickest at the insertion of the pectoralis major on the humerus and thinnest toward its caudal edge. This can be visualized particularly well when performing a trans-axillary augmentation. However, if electrocautery is used for dissection of this plane, the delicate fascia can disintegrate with heat. Yes, there is marginally greater thickness on the more cephalad location to the humeral and clavicular insertion. However, how often do we dissect the entire pectoralis major for breast augmentation? Gone are the days of large pockets, especially when working with more cohesive implants that require tight pockets to shape the breast.

When creating a “subfascial” pocket, is it really subfascial when part of the fascia disintegrates as it is separated from the pectoralis major? Or does the dissection inadvertently include some of the serratus fascia to truly support an implant in the “subfascial” plane?

We have all seen the pectoral fascia and know its thickness is questionable. Even for a skilled, senior surgeon, it is difficult to know with certainty when we are truly subfascial, particularly inferiorly at the start of an IMF dissection. What exactly are we gaining with the addition of a mere millimeter of tissue to the anterior aspect of the implant pocket? This begs the question: *is “subfascial” a misnomer?* Given the minimal thickness and coverage provided by the pectoral fascia, we can rarely guarantee any given dissection is completely subfascial or subglandular, and thus we posit that both “subfascial” and “subglandular” exist rather under the prepectoral umbrella term.
